# Co-Stimulation of Oxytocin and Arginine-Vasopressin Receptors Affect Hypothalamic Neurospheroid Size

**DOI:** 10.3390/ijms22168464

**Published:** 2021-08-06

**Authors:** Mohammad Saied Salehi, Inga D. Neumann, Benjamin Jurek, Sareh Pandamooz

**Affiliations:** 1 Clinical Neurology Research Center, Shiraz University of Medical Science, Shiraz 7193635899, Iran; saied.salehi@gmail.com; 2 Department of Molecular and Behavioural Neurobiology, Institute of Zoology, University of Regensburg, 93051 Regensburg, Germany; inga.neumann@ur.de; 3 Institute for Molecular and Cellular Anatomy, University of Regensburg, 93051 Regensburg, Germany; 4 Stem Cells Technology Research Center, Shiraz University of Medical Sciences, Shiraz 7193635899, Iran

**Keywords:** oxytocin, spheroid, proliferation, OXTR, V1aR, cytoskeleton

## Abstract

Oxytocin (OXT) is a neuropeptide involved in a plethora of behavioral and physiological processes. However, there is a prominent lack of 3D cell culture models that investigate the effects of OXT on a cellular/molecular level. In this study, we established a hypothalamic neuronal spheroid model to investigate the cellular response in a more realistic 3D setting. Our data indicate that the formation of spheroids itself does not alter the basic characteristics of the cell line and that markers of cellular morphology and connectivity are stably expressed. We found that both OXT and arginine vasopressin (AVP) treatment increase spheroid size (surface area and volume), as well as individual nucleus size, which serves as an indicator for cellular proliferation. The cellular response to both OXT and AVP seems mainly to be mediated by the AVP receptor 1a (V1aR); however, the OXT receptor (OXTR) contributes significantly to the observed proliferative effect. When we blocked the OXTR pharmacologically or knocked down the OXTR by siRNA, the OXT- or AVP-induced cellular proliferation decreased. In summary, we established a 3D cell culture model of the neuronal response to OXT and AVP and found that spheroids react to the treatment via their respective receptors but also via cross-talk between the two receptor types.

## 1. Introduction

The neuropeptide oxytocin (OXT) is not only involved in the classical neuroendocrine regulation of physiological processes such as milk letdown or labor but also of various social and emotional behaviors such as maternal behavior, anxiety, or social motivation [1,2,3]. We previously showed on a cellular level that OXT regulates neuronal morphology, ATP production, and calcium signaling [4,5], which are processes that regulate neuronal connectivity. Neuronal connectivity can be compromised in autism spectrum disorder (ASD), which might explain why some ASD patients benefit from intranasal OXT treatment [6]. The OXT system is highly cross-linked with other neuropeptide systems; for instance, we previously showed that intranasal OXT treatment affects the GnRH, kisspeptin, and neurokinin B systems [7,8]. Additionally, OXT modulates the anxiogenic activity of the corticotropin-releasing factor system [9,10,11] and interacts with the neuropeptide S system [12]. Moreover, the arginine vasopressin (AVP) system, which is evolutionarily related to the OXT system [13,14], shows cross-talk with the OXT system in a variety of different contexts, such as female aggression [15], substance use disorder [16,17], or ASD and schizophrenia [18]. Depending on the context and readout parameter, OXT and AVP can act in a concerted agonistic manner or show oppositional antagonistic features. For instance, AVP has been shown to act antiapoptotic in the hypothalamic rat cell line H32 [19] and hippocampal primary neurons [20]. We detected a similar antiapoptotic response of the same cell line, H32 cells, to various concentrations of OXT and the selective agonist TGOT and confirmed the positive effect of AVP on cell viability [5]. Moreover, OXT and AVP exert similar effects on cellular morphology, i.e., neurite outgrowth or retraction, via their respective receptors [4,5,21].

This intracellular cross-talk is possible, as subpopulations of OXT/AVP receptor-coexpressing neurons in the PVN and BNST [22], hippocampus [23], cortex [24], or peripheral mononuclear blood cells [25] have been detected. Although OXT has been thoroughly studied in vivo and in multiple in vitro systems over the past decades [1,26], 3D cell culture has rarely been adopted. This has led to a confusing amount of contradicting cellular responses to OXT (e.g., regarding proliferation [27]), which are, to a certain extent, very likely caused by diverging cell culture treatment conditions and artifacts, in addition to the inherent biological promiscuity of the OXT receptor (OXTR) system itself [28]. This promiscuity has been studied in detail in 2D cell culture systems, e.g., the coupling of Gi, Go, or Gq proteins to the receptor, the optional recruitment of β-arrestin and subsequent receptor-desensitization and internalization [29], or the interplay with other receptors, such as β-2 adrenergic receptors [30] or AVP receptors (V1aR, V2 [31,32]). Meanwhile, it has been widely accepted that 3D culture of primary cells or cell lines recreates the natural cellular environment with superior accuracy [33,34,35]. Moreover, researchers realized recently that the characteristics of the extracellular matrix (ECM), i.e., the attachment surface of cell culture dishes, influences the transcriptome of the cultured cells [36]. For instance, we showed in a recent study that epidermal neural crest stem cells react to the stiffness of the ECM [37] with altered morphology and gene expression of growth factors. In addition, OXT can induce epithelial–mesenchymal transition (EMT) of cell lines [38], thereby completely changing cellular characteristics such as morphology, polarity, and the transcriptome. EMTs occur mainly in epithelial cells that detach from their tissue of origin, for instance in tumor cells that disseminate from the primary tumor and form metastases [39]; however, an EMT can also be induced by spheroid formation [40]. Therefore, recreating a natural 3D environment for neuronal cells and, at the same time, closely monitoring the cellular characteristics is of utmost importance.

Consequently, we assessed the potential of a well-described OXTR/V1aR cell line, the rat hypothalamic neuronal cell line H32 [5,9,19,41], to form spheroids and to characterize the cellular response to OXT in 3D. In addition, we asked whether OXT-induced cellular effects originated from OXTR-, V1aR-, or combined receptor activation. Therefore, we applied a multitude of different pharmacological agonists/antagonists and supported the data with an siRNA-mediated knockdown approach.

## 2. Results

### 2.1. Spheroid Formation

**Figure 1:**
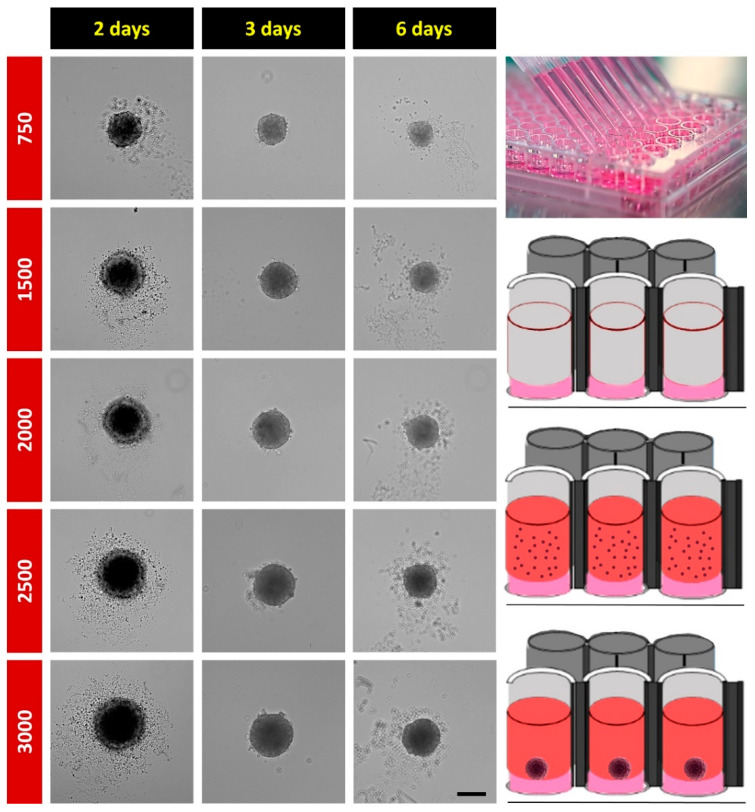
Left panel: bright-field images of H32 neurospheroids with 750, 1500, 2000, 2500, or 3000 initial cells following 2-, 3-, and 6-day in vitro cultures. On Day 3, the spheroids were fully formed and started to decay from there on. Therefore, all experiments were conducted within 3 days. Scale bar: 100 µm; right panel: schematic experimental outline of the spheroid formation.

To create an optimal 3D cell culture model that allows studying the effects of OXT on cellular processes in a natural 3D environment, we first tested whether the hypothalamic neuronal cell line H32 is able to generate spheroids. To generate hypothalamic neurospheroids, 750, 1500, 2000, 2500, or 3000 H32 cells were seeded on 1.5% agarose-coated 96-well plates, and spheroids were monitored for up to 6 days. As shown in Figure 1, two days after seeding, H32 cells formed round, compact spheroids; however, a significant number of unattached cells remained around the main core. Unattached cells disappeared on Day 3, and spheroid size was at maximum but gradually decreased afterwards. Consequently, we chose 3000 cells as the initial seeding cell number and a three-day culturing/treatment regimen for further experiments.

### 2.2. Marker Expression in H32 Neurospheroids

**Figure 2:**
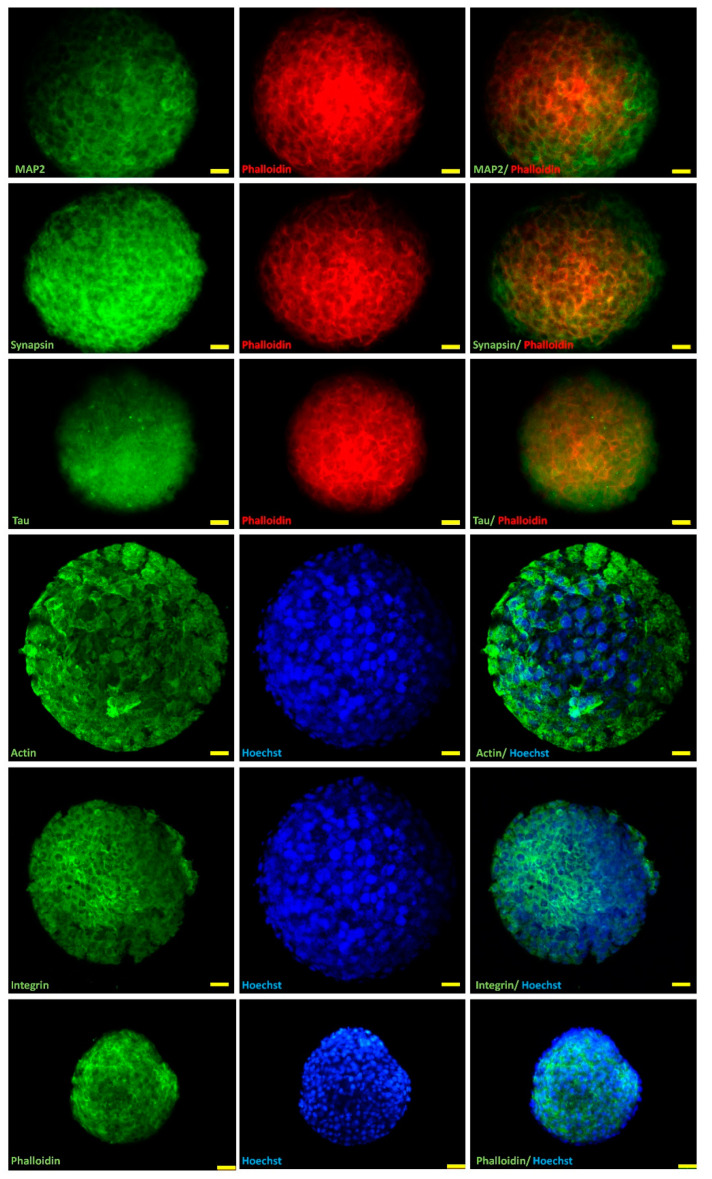
Confocal images of 3-day-old H32 neurospheroids stained against MAP2, synapsin1/2, tau, beta actin, beta integrin, and F-actin/Phalloidin (all marked with Alexa488/green) as markers for synaptogenesis, dendrites, axons, and the cytoskeleton. Where necessary Phalloidin is marked red, and nuclei were stained using Hoechst 33,342 (blue). Scale bar is 25 µm.

Cell lines whose culture parameters switch from 2D to 3D can undergo epithelial–mesenchymal transition (EMT)-like processes [40,42,43]. To characterize the 3D-grown neurons and visualize the expression of key markers of OXT-induced neuronal morphology and connectivity in H32 spheroids, neurospheroids were immunostained for microtubule-associated protein 2 (MAP2) for dendrite and perikarya staining, tau for dendrite, perikarya and axon staining, synapsin1/2 for dendritogenesis, and β-integrin for cell adhesion and ECM interaction. Furthermore, to visualize alterations in cytoskeletal arrangement, spheroids were stained for monomeric beta actin (antibody staining) and filamentous F-actin (phalloidin staining). The images obtained indicate a uniform and stable expression level of the neuronal/cellular markers in 3-day-old hypothalamic neurospheroids (Figure 2), indicating a stable neuronal cell type with no obvious morphological alterations, despite 3D culturing conditions. Actin fiber formation was visualized by staining globular (beta)-actin and filamentous F-actin. The stable presence of F-actin negates the notion of a possible epithelial–mesenchymal transition (EMT), as an EMT would cause cytoskeletal rearrangements into thick stress-fibers and mainly microtubule-based protrusions [44]. EMTs disrupt intercellular signaling and alter the interaction with the extracellular matrix [44]. Synapsin expression indicates normal intercellular signaling, and integrin expression is a rough indicator of interactions with the extracellular matrix.

### 2.3. OXT and AVP Dose Dependently Increase H32 Neurospheroid and Nucleus Size

**Figure 3:**
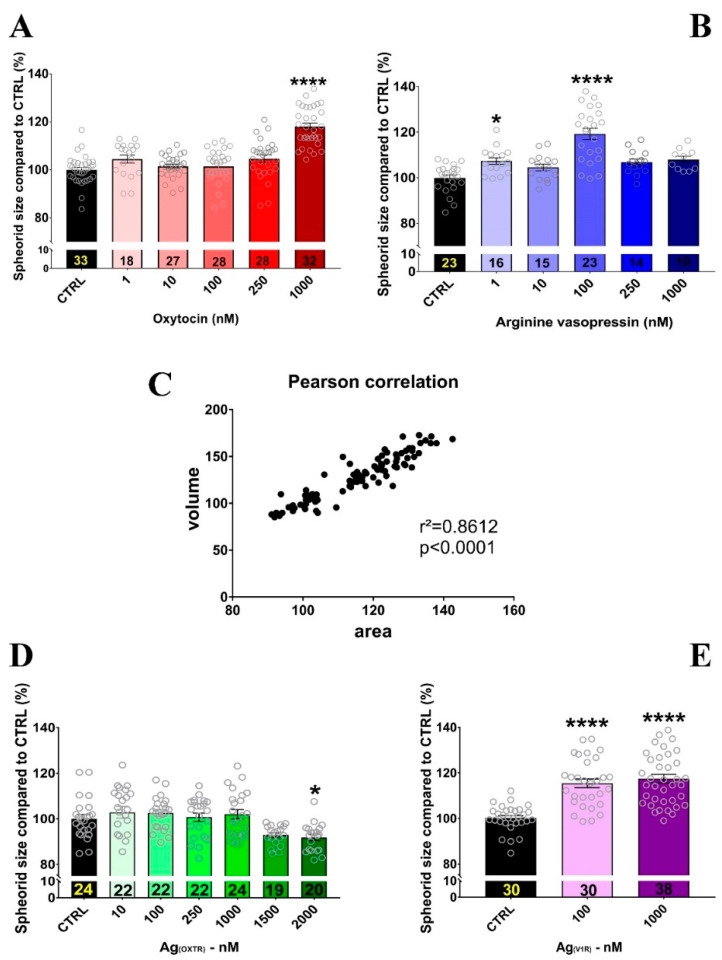
Effects of a 3-day treatment regimen of OXT, AVP, and their cognate receptor agonists on H32 neurospheroid size. (**A**) Only 1000 nM OXT was effective in significantly increasing spheroid size by 118%. One-way ANOVA (F_(5,160)_ = 28.53, *p* < 0.0001); Holm–Sidak **** *p* < 0.0001 compared to the control (CTRL) group. (**B**) AVP increased spheroid size at 1 nM and 100 nM, but not 10, 250, or 1000 nM. One-way ANOVA (F_(5,95)_ = 16.38, *p* < 0.0001); Holm–Sidak * *p* < 0.05 and **** *p* < 0.0001 compared to CTRL. (**C**) The measured spheroid volume was significantly and positively correlated with the surface area of the spheroids (r^2^ = 0.8612, *p* < 0.0001), indicating a positive effect of OXTR and V1aR on spheroid size and surface area. (**D**) Treatment with the OXTR agonist TGOT (Ag_(OXTR)_) reduced spheroid size at 2000 nM. One-way ANOVA (F_(6,146)_ = 5840, *p* < 0.0001); Holm–Sidak * *p* = 0.0317 versus CTRL. (**E**) Treatment with a vasopressin receptor agonist (Ag_(V1R)_) at 100 and 1000 nM increased spheroid size. One-way ANOVA (F_(2,95)_ = 29.07, *p* < 0.0001); Holm–Sidak **** *p* < 0.0001 compared to CTRL.

To evaluate the effects of OXT and AVP, hypothalamic neurospheroids were treated with 1, 10, 100, 250, and 1000 nM of each peptide. Spheroid size was measured on Day 3 of treatment. As shown in Figure 3A, 1000 nM OXT increased spheroid surface area up to 18%; however, lower concentrations were not effective. On the contrary, all doses of AVP, except 10 nM, increased the neurospheroid area, with 100 nM AVP exerting the largest effect size (Figure 3B). The spheroid volume correlated significantly with the surface area of the spheroids (r^2^ = 0.8612, *p* < 0.0001; Figure 3C).

Because both OXT and AVP can bind to each other’s receptors, we used receptor agonists to assess the specificity of the observed effect. V1b expression has not been detected in H32 cells [5] and is, therefore, disregarded in this study. Surprisingly, 10 to 1500 nM of the OXTR agonist TGOT was ineffective in increasing the neurospheroid size, and 2000 nM even decreased spheroid size (Figure 3D). The higher doses compared to OXT were chosen based on the lower affinity for the OXTR (Ki OXT => OXTR = 0.83 nM; TGOT => OXTR = 0.04 nM) [45]. In contrast, the Ag_(V1aR)_ at 100 and 1000 nM increased the spheroid size (Figure 3E), suggesting that changes in spheroid size under OXT or AVP treatment are mainly caused by the V1aR, and is not related to OXTR activation.

**Figure 4:**
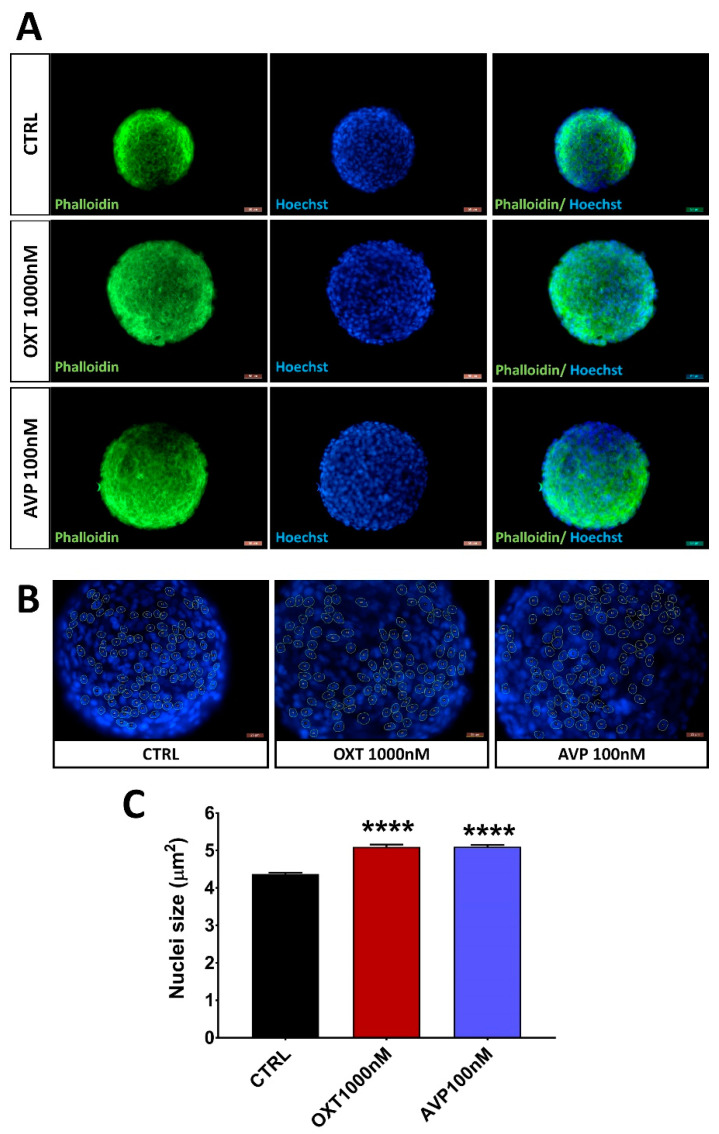
(**A**) Representative confocal images of 3-day-old H32 neurospheroids treated with either H_2_O (control), 1000 nM OXT, or 100 nM AVP for 3 days. Phalloidin is marked green and Hoechst 3334s is marked blue. (**B**)Visualization of the quantification of nucleus size in a control, OXT, or AVP treated spheroid. Yellow circles indicate analyzed nuclei within that spheroid. Scale bars represent 50 µM. (**C**) Quantification of nucleus size by Hoechst 33,342 staining and shape detection. Nucleus size increased from 4.3 to 5.064 and 5.100 µm^2^ (n = 100 F_(2,296)_ = 83.26, *p* = 0.0001, Holm–Sidak **** *p* < 0.0001).

Furthermore, Hoechst 33,342 counterstained nuclei of H32 neurospheroids treated with either 1000 nM OXT or 100 nM AVP with both treatments also significantly increased the nucleus size (Figure 4).

Considering that those receptor agonists are not 100% receptor specific [46], we sought to fully disentangle the contribution of both receptors to the spheroid growth by combining either the peptide or an agonist with the corresponding antagonist.

### 2.4. Partial Blockade of OXTR and V1aR by Antagonists Suggests a Role for Both Receptors in the Regulation of Spheroid Size

**Figure 5:**
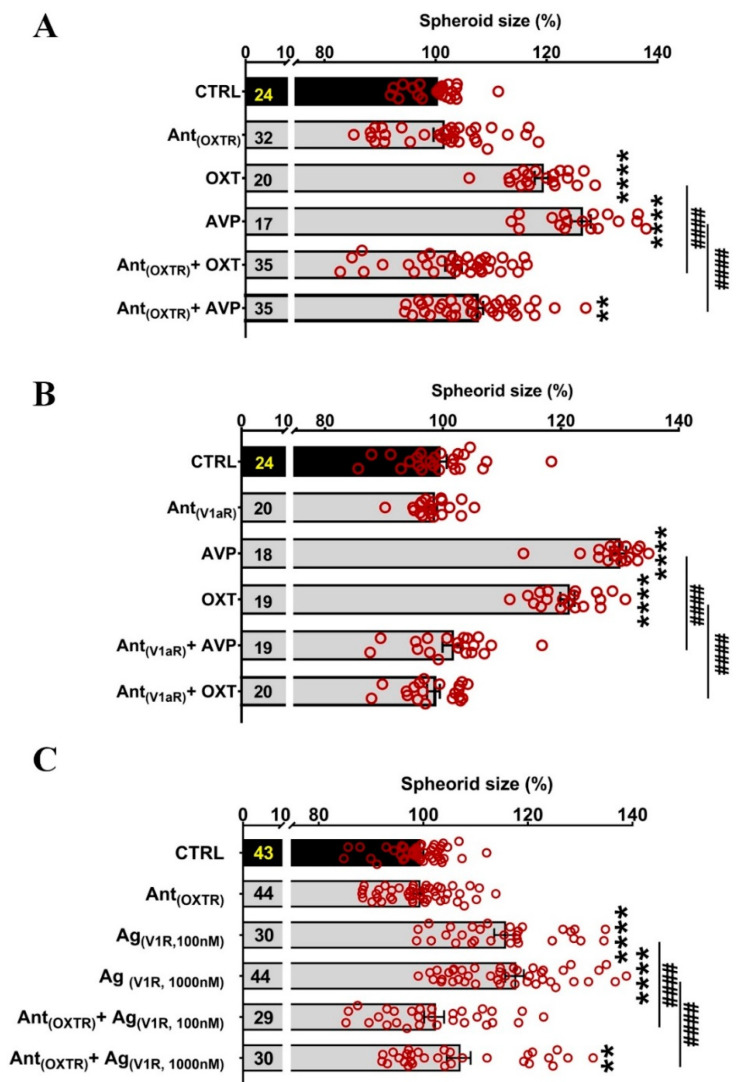
Effects of the Ant_(OXTR)_ and Ant_(V1R)_ on the inhibition of OXT/AVP-induced neurospheroid size changes. (**A**) OXT and AVP both increase spheroid size significantly. The Ant_(OXTR)_ blocks the OXT- and AVP-induced increase. One-way ANOVA (F_(5,156)_ = 39.18, *p* = 0.0001), Holm–Sidak ** *p* = 0.005, **** *p* < 0.0001 compared to CTRL; #### *p* < 0.0001 compared to the indicated group. (**B**) Similar to the Ant_(OXTR)_, the Ant_(V1R)_ blocks the OXT- and AVP-induced increase in spheroid size. One-way ANOVA (F_(5,113)_ = 124.5, *p* < 0.0001); Holm–Sidak **** *p* = 0.0001 compared to CTRL; **####** *p* = 0.0001 compared to the indicated group. (**C**) The Ag_(V1R)_ increases spheroid size to a similar extent as OXT and AVP, however, the effect is blocked by the Ant_(OXTR)_. One-way ANOVA (F_(5,214)_ = 28.16, *p* < 0.0001), Holm–Sidak ** *p* = 0.005, **** *p* = 0.0001 compared to CTRL; #### *p* = 0.0001 compared to the indicated group.

To further investigate the contribution of the OXTR and V1aR to the observed increase in neurospheroid size, we also employed OXTR and V1aR antagonists (Ant_(OXTR)_, Ant_(V1aR)_). The Ant_(OXTR)_ was able to fully inhibit the oxytocin effect; however, it also partially attenuated the AVP impact (Figure 5A). On the contrary, the Ant_(V1aR)_ prevented both OXT and AVP effects (Figure 5B). These data could suggest an exclusive V1aR-mediated effect; however, when the Ant_(OXTR)_ was applied, the effect of the Ag_(V1aR)_, especially at the dose of 100 nM, was also fully blocked (Figure 5C). This, in turn, indicates that either the Ag_(V1R)_ is not specific for the V1aR or the Ant_(OXTR)_ is unspecific for the OXTR. Either possibility also implicates a potential combinatorial effect of both receptors to the effect.

### 2.5. Effects of OXTR and V1aR Knockdown on Neurospheroid Size Changes

**Figure 6:**
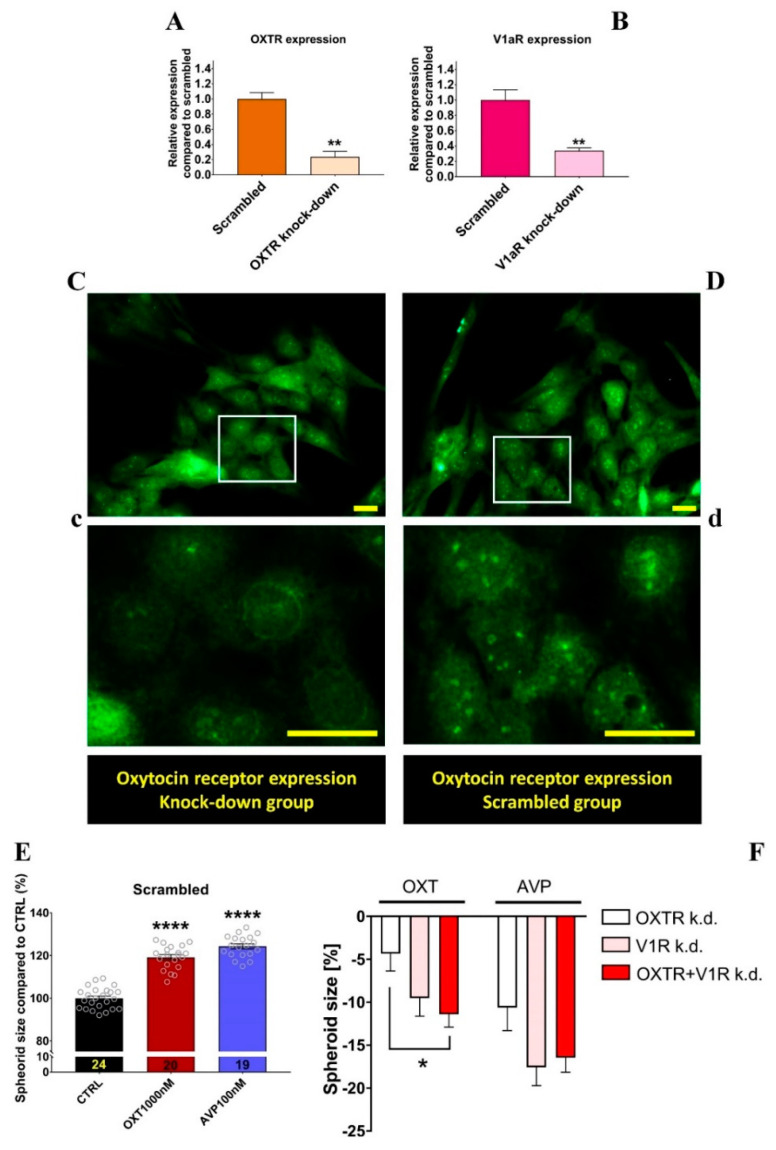
(**A**) Relative mRNA expression levels of the OXTR in H32 cells transfected with OXTR siRNA. OXTR mRNA levels were downregulated to 20%. ** *p* = 0.01 (**B**) Relative mRNA expression of V1aR in H32 cells transfected with V1aR siRNA decreased expression to 30%. ** *p* = 0.01 (**C**) Protein expression of the OXTR in H32 cells transfected with OXTR siRNA and (**D**) scrambled siRNA. (**c**,**d**) are the corresponding magnifications. Scale bar represents 25 µm. (**E**) Effects of OXT and AVP on the scrambled siRNA transfected H32 spheroid size. **** *p* = 0.001 (**F**) Inhibitory effect of OXTR, V1aR, and combined knockdown of both receptors on OXT- and AVP-induced spheroid size increase. Values depict reduced spheroid size by indicated treatment, relative to the respective scrambled siRNA treated group. Knockdown of the OXTR reduces OXT-induced spheroid growth by 5% and AVP-induced growth by 10%. Combined knockdown of both receptors significantly reduced spheroid growth by 12% compared to just OXTR knockdown. One-way ANOVA (F_(2,55)_ = 3.650, *p* = 0.0325); Holm–Sidak * *p* = 0.0333, n = 18–20.

As pharmacological inhibitors lack absolute specificity, we confirmed our pharmacological data by siRNA-mediated knockdown of the OXTR and V1aR. Knockdown efficiency was tested 40 h after transfection and was found to reduce the mRNA expression of the respective receptor by 80% (Figure 6A,B). Protein knockdown was evaluated 3 days after transfection. Immunostainings clearly demonstrated lower levels of OXTR in the knockdown group (Figure 6C) compared to scrambled siRNA-transfected cells (Figure 6D). Despite earlier negative reports on the specificity of commercially available OXTR antibodies [47], we showed in a previous publication [10] that the alomone antibody is indeed specific for the rat OXTR in IHC.

When transfected with scrambled siRNA, H32 cells showed the expected increase in spheroid size upon OXT and AVP treatment (Figure 6E). We then measured the size of target-specific siRNA-transfected spheroids, which also received OXT or AVP treatment. We then calculated the delta percentage between scrambled and target siRNA groups, which corresponds to the level of growth inhibition.

When transfected with OXTR-specific siRNA, the OXT-induced spheroid growth was reduced by only 4%. Knockdown of V1aR was twice as effective and reduced OXT-induced growth by 10%. By knocking down both receptors simultaneously, we detected a combinatorial effect that added up to 12% compared to just OXTR knockdown.

In contrast, all knockdowns were equally effective in reducing the growth of AVP-treated spheroids. The fact that an OXTR knockdown reduces AVP-induced effects and a V1aR knockdown reduces OXT-induced effects again indicates a combinatorial effect of both receptors in regulating spheroid growth.

## 3. Discussion

The role of endogenous and synthetic OXT and AVP in physiological and behavioral processes has been studied extensively during previous decades. In particular, the effects of OXT applied via various routes, e.g., intranasal, intravenous, or intracerebroventricular, have been investigated on a behavioral and cellular level [1,48,49]. However, the use of receptor-specific antagonists and agonists has only partially been adopted in human and animal studies or 2D cell culture experiments [48,50,51]. Dissecting the cross-talk between OXTR and V1aR, however, is of paramount importance, as both peptides bind each other’s receptors with only slightly different receptor affinities [29]. Moreover, the cellular effects of OXT and AVP under 3D cell culture conditions have rarely been investigated [52,53]. The formation of spheroids mimics the 3D arrangement of cells in any given tissue, and spheroid size or volume is frequently used as a proxy for cellular proliferation under 3D conditions [54].

In this study, we demonstrated the combinatorial OXTR- and V1aR-mediated effects on spheroid size (surface area and volume)/cellular proliferation in a 3D culture of hypothalamic neurons. We found that subjecting spheroids to high doses of OXT increases spheroid size, while lower doses are not effective.

AVP in various doses was more effective in increasing spheroid size, so one could conclude that the AVP system solely regulates cell proliferation and that high doses of OXT only transactivate the V1aR. As the V1bR is not expressed in H32 cells, we refrain from speculating about a potential OXTR-V1bR cross-talk.

However, pharmacological blockade of either the OXTR or the V1aR, as well as exclusive receptor activation by specific agonists, indicates a concerted role of both receptors in the observed effect on spheroid size.

Taking into consideration that pharmacological agonists and antagonists may not be fully receptor specific [46], we adopted an siRNA approach where we knocked down either one or both receptors. Those results support the notion that both receptors work in conjunction to bring about the proliferative effects in spheroids. Both receptors unexpectedly increased spheroid size, with AVP exerting larger effect sizes than OXT. Taking into consideration other known examples of cross-talk between different GPCRs [55,56,57], it is tempting to hypothesize that the OXTR and V1aR act in concert and might even form heterodimeric units that unilaterally affect cellular proliferation. Although this hypothesis needs further verification in future studies, OXTR and V1aR cross-talk and its effects on behavior and physiology have been thoroughly discussed and nicely summarized by Song and Albers [58].

To strengthen the validity of our data, we did not only assess the size of the whole spheroid but also the size of the individual nuclei as a proxy for proliferation. Proliferating cells enlarge their nucleus in order to prepare for cell division [59]; consequently, enlarged nuclei by OXT and AVP treatment indicate a prominent role for both peptides in cellular proliferation in a 3D culture.

Cellular proliferation aligns with other measurable cellular functions, such as increased metabolism of provided substrates (referred to as cellular viability), mitochondrial ATP production, and the formation or retraction of cellular processes (e.g., dendritogenesis [60]). We previously showed that those cellular functions are initiated and regulated by the activation of the OXTR in conventional 2D cell cultures [4,5].

As pointed out by numerous studies [33,34,35,40,61], 3D culturing of cells is superior to 2D culture, as it allows a natural intercellular communication and eliminates any artificial surface area (plastic dishes, etc.) that can affect intracellular pathways via extracellular linker proteins such as β-integrin. The α- and β-integrin receptors form transmembrane heterodimers that functionally link the extracellular matrix with cytoskeletal actin proteins [62]. Integrins mediate cell signaling, which regulates multiple endothelial cell functions, including proliferation, cell viability, migration, differentiation, adhesion, and morphology [63]. Moreover, oxytocin induces contraction of cultured mammary myoepithelial cells via activated β-integrins [62]. Cell contraction relies on the formation of cytoskeletal filamentous actin, or F-actin, which can be visualized by phalloidin. The polymerization of globular actin, in our case visualized by the β-actin antibody, into F-actin is one sign of cytoskeletal rearrangements and precedes morphological alterations. Morphological alterations in neurons consist of neurite outgrowth or retraction. We [4,5] and others [64,65] previously showed that OXT induces morphological alterations in neurons and glia cells in a conventional 2D cell culture. With this study, we hope to prompt future studies that use 3D spheroid models to validate those 2D-derived findings.

In summary, our study used a 3D spheroid model to investigate OXTR- and V1aR-mediated cellular effects, alone and in combination. We made use of a spheroid culture model, as the natural 3D environment provides more realistic features of in vivo tissue. We found that high doses of OXT and lower doses of AVP drive cellular proliferation via reciprocal cross-talk between the OXTR and the V1aR.

## 4. Materials and Methods

### 4.1. H32 Culture and Spheroid Formation

The immortalized rat hypothalamic cell line (H32 cells, passage 15–30) was cultured in DMEM/F12 supplemented with 10% fetal bovine serum and 1% penicillin/streptomycin (growth medium). Cells were maintained in a humidified atmosphere containing 5% CO_2_ at 37 °C and passaged twice a week by gentle trypsinization. To form neurospheroids, first, 1.5% agarose (#A9539, Sigma) was prepared in serum-free DMEM/F12 and sterilized by autoclave (20 min, 121 °C, 1 bar). Then, 50 µL of hot agarose was quickly added to each well of a sterile flat-bottomed 96-well plate. After agarose polymerization, H32 cells ranging from 750 to 3000 cells/200 µL of growth medium were seeded onto agarose-coated 96-well plates and incubated for the desired time without any movement.

### 4.2. H32 Neurospheroid Treatment

Immediately after cell seeding onto agarose-coated plates, H32 cells were treated with OXT (1, 10, 100, 250, 1000 nM; #4016373, Bachem, Bubendorf, Switzerland), AVP (1, 10, 100, 250, 1000 nM; #2935, Tocris, Bristol, UK), OXTR agonist TGOT (10, 100, 250, 1000, 1500, 2000 nM; #4013837, Bachem), or V1R agonist oxytocin trifluoroacetate salt (100, 1000 nM; #4030889, Bachem). OXTR antagonist L-371,257 (1 mM; #2410, Tocris) or V1aR antagonist Manning compound (1 mM, kindly provided by M. Manning) was applied 30 min before the treatments.

### 4.3. H32 Neurospheroid Staining

H32 neurospheroids were stained by immunohistochemistry (IHC). Briefly, neurospheroids were washed with PBS and fixated using 4% paraformaldehyde or absolute methanol, depending on the primary antibody. Following additional washing steps, spheroids were blocked in 10% normal goat serum or 1% bovine serum albumin containing 0.3% Triton X100. Then, neurospheroids were incubated overnight at 4 °C with primary antibodies as follows: chicken anti-MAP2 (#188006, Synaptic Systems, Göttingen, Germany), guinea pig anti-Synapsin 1/2 (#106004, Synaptic Systems), guinea pig anti-Tau (#314004, Synaptic Systems), mouse anti-beta actin (#3700s, Cell Signaling, Danvers, MA, USA) or goat anti-Integrin beta1 (#sc6622, Santa Cruz, Dallas, TX, USA). After 24h, spheroids were washed with PBS and incubated with the compatible secondary antibody: goat anti-chicken Alexa Fluor 488 (#A11039, Invitrogen, Carlsbad, CA, USA), goat anti-guinea pig Alexa Fluor 488 (#A11073, Invitrogen), goat anti-mouse Alexa Fluor 488 (#A11001, Invitrogen), or rabbit anti-goat Alexa Fluor 488 (#A11078, Invitrogen). Spheroids were also incubated with Phalloidin Alexa Fluor 488 (#8878, Cell Signaling) or Phalloidin iFluor 594 (#ab176757, Abcam, Cambridge, UK) diluted in blocking solution for 1 h. Cell nuclei were counterstained with Hoechst 33,342 (#H1399, Thermo Fisher, Waltham, MA, USA).

### 4.4. OXTR and V1aR Knockdown

H32 cells were transfected with three unique 27mer rat OXTR siRNA duplexes (#SR504035, Origene, Rockville, MD, USA) and/or three unique 27mer rat V1aR siRNA duplexes (#SR502551, Origene) for the knockdown of OXTR and/or V1aR. The Neon Transfection System (Thermo Fisher; MPK5000, Waltham, MA, USA) was employed for this purpose. Briefly, at each transfection point, 10^5^ cells were resuspended in 10 µL of buffer R, and three duplexes of each siRNA were mixed 1:1 and added for transfection at the final concentration of 30 nM. The electroporation settings were 900 V, 35 ms, and 3 pulses. Transfected cells were seeded onto agarose-coated 96-well plates without antibiotics and treated with OXT or AVP, as described earlier. To verify the specificity of the knockdown, the universal scrambled negative control siRNA duplex (#SR30004, Origene) was used.

### 4.5. Quantification of Transfection Efficiency

To screen the efficiency of transfection, the OXTR and V1aR mRNAs were evaluated in 2D-cultured H32 cells 48 h after transfection. To do so, total RNA was extracted using the RNeasy Mini Kit (#74104, Qiagen, Hilden, Germany). To eliminate genomic DNA contamination, on-column DNase digestion was performed with the RNAse-Free DNase Set (#79254, Qiagen). The cDNA was synthesized by the SuperScript IV First-Strand Synthesis System (#18091050, Invitrogen) according to the manufacturer’s instructions. The qRT-PCR run was performed on the QuantStudio 3 Real-Time PCR System (Thermo Fisher) with the following amplification conditions: 50 °C for 2 min, 95 °C for 2 min, and then 40 cycles of 95 °C for 3 s and 60 °C for 30 s. Each reaction contained a first-strand cDNA template, specific primer sets (OXTR forward: 5′-CTGGAGTGTCGAGTTGGACC-3′, OXTR reverse: 5′-AGCCAGGAACAGAATGAGGC-3′ or V1aR forward: 5′-ACTGTGAAGATGACCTTTGTG-3′, V1aR reverse: 5′-GAATCGGTCCAGATGAAATTCTC-3′), and PowerUp SYBR Green Master Mix (#A25743, Thermo Fisher). Finally, the relative expressions of OXTR and V1aR in the knockdown cells compared to the scrambled were calculated using the ΔΔct method.

The expression of the OXTR at the protein level was also evaluated in 2D-cultured H32 cells 3 days after the transfection by performing immunocytochemistry (ICC). In brief, cells were fixated with 4% paraformaldehyde and washed by PBS. After blocking with 1% fetal bovine serum, 1% normal goat serum, and 0.1% Triton X100, cells were incubated with rabbit anti-OXTR polyclonal antibody (#AVR-013, Alomone Labs, Jerusalem, Israel) diluted in PBS containing 0.5% TritonX-100 and 3.3% fetal bovine serum overnight at 4 °C. The next following day, the second blocking step in 3% bovine serum albumin and goat anti-rabbit Alexa Fluor 488 (#A11304, Thermo Fisher) secondary antibody was applied. Finally, the slides were covered with the ProLong Glass Antifade Mountant (#P36984, Invitrogen).

### 4.6. Imaging and Spheroid Size Measurement

The H32 neurospheroid pictures were taken using a ZOE Fluorescence microscope (Bio-Rad, Hercules, CA, USA). Stained spheroid pictures were taken on an AiryDisc confocal laser-scanning microscope (Zeiss, Jena, Germany). The 2D-cultured H32 pictures were taken by a fluorescence inverted microscope (Leica, Wetzlar, Germany). The surface area of H32 neurospheroids was measured by calculating the area of spheroids using ImageJ software (Fiji version, 1.52p, used 1 December 2019 to 15 February 2020 NIH, Bethesda, MD, USA). In addition to the surface area, we analyzed the volume of the spheroids by calculating it from two diameters and the formula V = 4/3π ((D1 + D2)/4)^3^.

### 4.7. Statistical Analysis

Parametric one-way (factor treatment) analysis of variance (ANOVA), followed by Holm–Sidak post hoc correction for multiple comparisons, was performed for statistical analyses (GraphPad Prism 8.0.2, San Diego, CA, USA, used 15 February 2020 to 1 July 2021). Outliers were identified and removed by the ROUT method (Q = 1%), and the data were tested for normal distribution by the Shapiro–Wilk and Kolmogorov–Smirnov tests. Statistical significance was accepted at *p* < 0.05. Data are presented as the mean ± standard error of the mean (SEM).

## Data Availability

Not applicable.
